# Parliament and the Professions

**Published:** 1921-04-30

**Authors:** 


### Parliament and the Professions.

### Charges Against Hospital.

Two questions were asked by Mr. N. Maclean as to the administration of the Bellahouston Hospital for discharged service men at Glasgow. It was alleged that 900 out-patients were examined in seven days and marked fit for work, and an instance was given of one of these collapsing the same day from heart disease. The Minister of Pensions said he had called for a report, and unless this satisfies him an inquiry will be made.

### Nurses' Registration Act.

In a question addressed to the Minister of Health by Mr. T. Griffiths, it was suggested that the General Nursing Council for Scotland has so far refused to unite with the English Council before making rules in respect of conditions of admission to the Register as required by the Scottish Act, and it was asked if steps will be taken to secure a consultation with the English Council in order to provide a uniform standard of qualification throughout the United Kingdom. Sir Alfred Mond said there had been no refusal to consult, and that the outstanding points were small in number. It had been suggested that the Conference should be deferred until the Draft Rules for the admission of future nurses are ready for discussion. In any case, although agreeing with the importance of a uniform standard, he had no jurisdiction over the Scottish Council. [A Conference between the three Councils was held on April 21, see next column.—Ed. The Hospital.]

In reply to a question by Mr. Grundy, the Minister of Health stated that the work of the General Nursing Council was not, as suggested, in the charge of a junior official in the Ministry of Health, but that all questions are dealt with by administrative and medical officers of appropriate standing, and subject to the Minister's instructions. The General Nursing Council have only been required to make such alterations in the Rules submitted by them as are necessary to bring them into conformity with the Act, or to give effect to the intention of Parliament.

With regard to the last points, a further question was asked by Mr. F. Hall. The General Nursing Council for England and Wales had submitted a rule purporting to give them a discretion to refuse to admit to their Register nurses already on the Scottish or Irish Registers. This, the Minister had been advised, is *ultra vires*, and he had been obliged to amend the rule in question. Subject to the submission of an amended rule to give effect to the reciprocity provisions of Section 6 of the Act, the Minister is prepared to sanction at once the rules for admission of existing nurses.

### Street Collections.

Viscount Curzon asked the Home Secretary a question about street collections, and the practice of the Chief Commissioner of the Metropolitan Police in granting permits for collections on behalf of charities in certain areas without reference to the local authorities concerned. Mr. Shortt replied that the Commissioner is under no obligation to consult the local authorities, but as a matter of courtesy he does so in cases of a local character. When, however, a collection is general in character it would not be practicable to consult the 135 authorities concerned, and the Commissioner decides the case on its merits after reference to the Advisory Committee appointed under the Regulations.

### Anatomical Study.

Sir H. Brittain asked the Minister of Health whether he is aware that there is the greatest difficulty in the proper prosecution of anatomical study in this country, resulting in a large number of students proceeding abroad to complete their studies. This, it was understood, was mainly due to the shortage in the supply of dead bodies for anatomical purposes. Sir Alfred Mond agreed as to the difficulty, but said that the state of affairs had been remedied by the action taken by his predecessor. There is still a shortage in the case of certain provincial medical schools, but this is being remedied.

